# Charter of the Kentucky State Dental Association

**Published:** 1870-04

**Authors:** 


### Proceedings of Societies.

### CHARTER OF THE KENTUCKY STATE DENTAL ASSOCIATION.

EDITOR REGISTER:—I send you our State Charter for publication. We tried a few years ago to get a law passed, but for want of attention it failed. So we thought we would get a State Charter for our society, and work under that a while, and probably it would be easier to get the law after a while. We will call a meeting in a short time to organize. Hope to be in working order before the American Dental Association meets in Nashville. W. H. SHADOAN.

### *An Act to incorporate the Kentucky State Dental Association.*

WHEREAS, Well-regulated dental societies have been found to contribute to the diffusion of true science, and the advancement of professional skill; and, whereas, certain persons, for the better promotion of these ends, desire a charter of incorporation, therefore,

SEC. 1. *Be it enacted by the General Assembly of the Commonwealth of Kentucky*, That the following persons, W. Muir Rogers, D D.S , W. G. Redman, D D.S., W. H. Shadoan, D.D.S., S. Driggs, D.D.S., A. S. Talbert, D.D.S. F. Peabody, D.D.S., G. S. Jones, D D.S , Samuel Griffith, D.D.S., together with their associates and successors, be, and they are hereby, created a body politic and corporate, by and under the name and style of the Kentucky State Dental Association; and by that name shall have perpetual succession, with power to adopt and use a common seal for the use of the same; to contract and be contracted with; to sue and be sued in all courts of law and equity in this State and elsewhere; to acquire by gift, grant, devise, purchase or otherwise, any real or personal property, and use, sell or dispose of the same at pleasure, in amount not to exceed $10,000 in value.

SEC. 2. The officers of this association shall be a President, Vice President, Recording Secretary, Corresponding Secretary, Treasurer and Librarian, whose term of office shall be one year, and until their successors shall be elected. In addition to the above officers, there shall be a board of three censors, elected by the association, who shall hold their office as follows: One for one year, one for two years, and one for three years, each of whose successors shall be elected for three years. The President and Recording Secretary shall be ex-officio members of the board of censors.

SEC. 3. This association shall have power to prescribe terms of qualification for the admission of members, adopt a constitution, by-laws and rules for its own government and that of its officers, not inconsistent with the laws of this State, or of those of the United States, and provide for amendments to their constitution and by-laws.

SEC. 4. This act shall be in force from and after its passage.

JNO. T. BUNCH,
Speaker of the House of Representatives.
P. H. LESLIE,
Speaker of the Senate.

By the Governor: J. W. STEVENSON.

SAM'L B. CHURCHILL, Sec'y of State.

COMMONWEALTH OF KENTUCKY—*SCT.*

I, Samuel B. Churchill, Secretary of State, do hereby certify that the foregoing bill is truly copied from the original enrolled bill on file in the office of the Secretary of State.

WITNESS my hand, and seal of State, at my office, in the city of Frankfort, Ky., this 21st day of February, 1870.

SAM'L B. CHURCHILL,
Secretary of State.

By W. T. SAMUELS, Ass't Sec'y.

